# The overexpression of major antioxidant enzymes does not extend the lifespan of mice

**DOI:** 10.1111/j.1474-9726.2008.00449.x

**Published:** 2008-11-25

**Authors:** Viviana I Pérez, Holly Van Remmen, Alex Bokov, Charles J Epstein, Jan Vijg, Arlan Richardson

**Affiliations:** 1 Barshop Institute for Longevity and Aging Studies, University of Texas Health Science Center at San AntonioSan Antonio, TX 78229, USA; 2 Department of Cellular and Structural Biology, University of Texas Health Science Center at San AntonioSan Antonio, TX 78229, USA; 3 Department of Physiology, University of Texas Health Science Center at San AntonioSan Antonio, TX 78229, USA; 4 Geriatric Research, Education, and Clinical Center, South Texas Veterans Health Care SystemSan Antonio, TX 78229, USA; 5 Institute of Human Genetics and University of CaliforniaSan Francisco, San Francisco, CA 94143, USA; 6 Department of Pediatrics, School of Medicine, University of CaliforniaSan Francisco, San Francisco, CA 94143, USA; 7 Department of Genetics, Albert Einstein College of MedicineBronx, New York, NY 10461, USA

**Keywords:** aging, antioxidant enzymes, transgenic and knockout mice

## Abstract

We evaluated the effect of overexpressing antioxidant enzymes on the lifespans of transgenic mice that overexpress copper zinc superoxide dismutase (CuZnSOD), catalase, or combinations of either CuZnSOD and catalase or CuZnSOD and manganese superoxide dismutase (MnSOD). Our results show that the overexpression of these major antioxidant enzymes, which are known to scavenge superoxide and hydrogen peroxide in the cytosolic and mitochondrial compartments, is insufficient to extend lifespan in mice.

The oxidative stress theory of aging offers a credible explanation of a molecular mechanism underlying the aging process. One of the most direct tests of the oxidative stress theory of aging has been to alter oxidative stress/damage and then determine how this alteration affects lifespan (e.g. to genetically manipulate the expression of antioxidant enzymes and observe the effects on lifespan). Based on studies to date, the effect of oxidative stress as a lifespan determinant has been dependent upon the type of animal model tested. Transgenic *Drosophila* overexpressing either CuZnSOD (Sun & Tower, 1999; Phillips *et al*., 2000) or MnSOD (Sun *et al*., 2002) have extended longevity.

Although several studies have shown that overexpression of antioxidant enzymes in mice has a protective effect against oxidative stress, with a diminished accumulation of oxidative damage in macromolecules (Muller *et al*., 2007), Huang *et al*. (2000) reported that transgenic mice overexpressing CuZnSOD (two- to five-fold increase) did not show any increase in lifespan (Huang *et al*., 2000). In contrast, Schriner *et al*. (2005) targeted catalase to mitochondria and observed a 21% extension in the lifespan of transgenic mice.

We studied the effects of CuZnSOD and catalase overexpression on lifespan, as well as the effects of combinations of CuZnSOD and catalase or CuZnSOD and MnSOD overexpression. Transgenic CuZnSOD and catalase mice were generated using large genomic segments of the human genes containing the intact genes with their endogenous promoter (Chen *et al*., 2003). The MnSOD transgenic mice were generated by Dr Epstein's laboratory using the mouse *Sod2* genomic fragment (Raineri *et al*., 2001). These mice overexpress the expected enzymes, with increases of two- to four-fold in all tissues tested and no diminution of the expression of other major antioxidant enzymes (Raineri *et al*., 2001; Chen *et al*., 2003; Mele *et al*., 2006). Fibroblast cell cultures derived from these mice were observed to be more resistant to oxidant stress (Mele *et al*., 2006; Shan *et al*., 2007).

Figure 1 shows the survival curves of each single or double transgenic mouse strain compared to the wild type (WT) controls. Analysis of the survival curves by the log-rank test (Andersen *et al*., 1993) showed no statistical differences in the survival curves between the WT mice and any of the transgenic mice. The survival data in Table 1 also show no significant differences in the mean, median, or 90% (when 90% of the mice have died) survivals of any of the transgenic strains compared to the WT mice. It should be noted that we studied cohorts of 44–54 animals, which allow us to detect a 10% change in mean survival (Liang *et al*., 2003). The mean and maximum survivals for the WT mice were more than 31 and 41 months (respectively), which is in the accepted range for well-maintained colonies of C57BL/6J mice and well-run longevity studies and is higher than published results for other vivaria (see Ran *et al*., 2007). Thus, the husbandry conditions used in this study minimize/eliminate deaths from infectious disease and other causes that can make survival data unreliable. Our data confirm the earlier study by Huang *et al*. (2000) showing that the overexpression of CuZnSOD has no effect on the lifespan of mice, but are strikingly different from what has been observed in *Drosophila* in which lifespan is increased when CuZnSOD is overexpressed (Sun & Tower, 1999; Phillips *et al*., 2000). Our data might appear to conflict with the study by Schriner *et al*. (2005), which showed that the overexpression of catalase increased the lifespan of transgenic mice. However, whereas Schriner *et al*. overexpressed catalase in mitochondria, catalase overexpression in our transgenic mouse occurred in the peroxisomes (Chen *et al*., 2003), where catalase is normally expressed (Zamocky & Koller, 1999).

**Table 1:** Statistical analysis of lifespan data for WT, CuZnSOD, catalase (CAT), CuZnSOD/CAT, and CuZnSOD/MnSOD transgenic mice

	WT	CuZnSOD-Tg	CAT-Tg	CuZnSOD/CAT-Tg	CuZnSOD/MnSOD-Tg
Number	44	44	44	47	54
Mean (days)	922	915	916	922.3	899
Median (days)	941	944	949	945	914
90% survival (days)	1090	1131	1099	1098	1075
Maximum (days)	1231	1229	1139	1163	1144

The survival data from Fig. 1 are expressed in days. Mean survivals (± standard error of the mean) for each group were compared to the WT group by performing a Student's *t*-test upon log-transformed survival times from the respective groups. The median and 90th percentile survivals for each group were compared to the WT group using a score test adapted from Wang *et al*. (2004). All the above comparisons were made individually between each experimental group (CuZnSOD-Tg, CAT-Tg, CuZnSOD/CAT-Tg, and CuZnSOD/MnSOD-Tg) and the WT control group; and Holm's method (Holm, 1979) was used to correct for multiple comparisons. None of the comparisons were significant, with *p*-values ranging from 0.19 to 0.97. After correcting for multiple comparisons, the *p*-values all became 1.

**Fig. 1:**
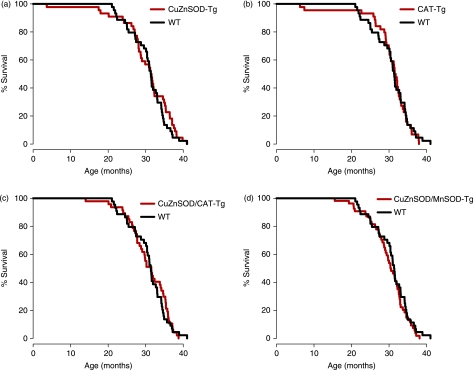
Lifespans of male transgenic mice overexpressing various antioxidant enzymes. Survival studies were conducted as described by Ran *et al*. (2007), and all mice were on the C57BL/6J background. The mice were housed four animals per cage starting at 2 months of age and fed a commercial mouse chow (Teklad Diet LM485) *ad libitum*. The survival of the WT cohort is presented in each of the graphs. The number of animals and the survival data are given in Table 1.

Our study is the first to determine whether simultaneously overexpressing two antioxidant enzymes has an effect on the lifespan of mice. Orr & Sohal (1994) initially reported that the overexpression of CuZnSOD and catalase together increased the lifespan of *Drosophila*. However, Tower's laboratory did not find a significant increase in the lifespan of *Drosophila* when either catalase (Sun & Tower, 1999) or MnSOD (Sun *et al*., 2004) was overexpressed with CuZnSOD. As shown in Fig. 1 and Table 1, overexpressing CuZnSOD and catalase or CuZnSOD and MnSOD had no effect on the lifespan of mice.

Based on the studies with *Drosophila*, we anticipated that we would see an increase in lifespan in one or more of the transgenic mouse models. However, our data demonstrate that overexpression of the major antioxidant enzymes that regulate oxygen metabolism in the cell, either by themselves or in combination, does not have an impact on the lifespan of mice. Therefore, these findings do not provide support for the role oxidative stress in the aging of mice.
